# Antibiotic use for common illnesses in children living with disability: a multi-country study across 42 low- and middle-income countries

**DOI:** 10.1016/j.eclinm.2025.103326

**Published:** 2025-07-02

**Authors:** Shengyue Qiu, Mingli Xu, Yuanyang Wu, Chaojie Liu, Xiying Li, Xinyi Yang, Haohai Xia, Ruonan Wang, Zishu Ma, Fanqian Meng, Xinping Zhang, Gordon Liu, Hannah Kuper, Shanquan Chen, Lianping Yang

**Affiliations:** a School of Public Health, Medical Division, Sun Yat-sen University, Guangzhou, China; b School of Politics and Public Administration, South China Normal University, Guangzhou, China; c School of Medicine and Health Management, Tongji Medical College, Huazhong University of Science and Technology, Wuhan, China; d School of Psychology and Public Health, La Trobe University, Melbourne, Australia; e National School of Development, Peking University, Beijing, China; f Institute for Global Health and Development, Peking University, Beijing, China; g International Centre for Evidence in Disability, Faculty of Epidemiology and Population Health, London School of Hygiene & Tropical Medicine, London, UK; h Sun Yat-sen Global Health Institute, Institute of State Governance, Sun Yat-sen University, Guangzhou, China

**Keywords:** Child illness, Disability, Antibiotics, Equity, Low- and middle-income countries

## Abstract

**Background:**

Approximately 240 million children worldwide are living with disabilities. Understanding the association between disability status and reported antibiotic use for common illnesses can help develop strategies to address the critical intersection of antimicrobial resistance (AMR) and disability.

**Methods:**

Data were collected from 42 low- and middle-income countries through the UNICEF-supported Multiple Indicator Cluster Survey (2017–2023). Disability status was assessed using the Washington Group-Child Functioning Module. Reported antibiotic use was measured by whether children with disabilities received antibiotic treatment for common childhood illnesses. Logistic regression models were applied to investigate the association between disability status and the prevalence of acute respiratory infection (ARI), diarrhea, and fever in the past two weeks, as well as reported antibiotic use for these illnesses. Analyses controlled for age, sex, place of residence, mother's education, the number of children under five in the household and country.

**Findings:**

The study included 301,857 children, 6.9% of whom were living with disabilities. Children with disabilities were more likely to experience common illnesses compared to those without disabilities: aOR = 1.78 (95% CI: 1.34–2.36) for ARI and aOR = 1.54 (95% CI: 1.22–1.96) for fever. The odds of antibiotic use among children with disabilities were comparable to those without disabilities: aOR = 1.13 (95% CI: 0.68–1.87) for ARI, aOR = 0.93 (95% CI: 0.64–1.36) for diarrhea, and aOR = 1.23 (95% CI: 0.81–1.86) for fever. This varied across countries, the lower-middle income countries had lower odds of reported antibiotic use for ARI and diarrhea (aOR = 0.85, 95% CI: 0.74–0.97, aOR = 0.78, 95% CI: 0.64–0.95, respectively). Lesotho, Iraq, Comoros and Honduras had higher odds of reported antibiotic use for children with disabilities, and in Pakistan where children with disabilities had lower odds of reported antibiotic use. Subgroup analyses showed that girls with disabilities were less likely to use antibiotics for diarrhea (aOR = 0.78, 95% CI: 0.63–0.96) compared to girls without disabilities. Similarly, girls with disabilities had lower odds of using antibiotics (aOR = 0.53, 95% CI: 0.29–0.98) compared to boys with disabilities. The associations also varied by impairment type, children with seeing, controlling behaviour or learning impairments are less likely to have reported antibiotic use.

**Interpretation:**

Children with disabilities are at a higher risk of developing common illnesses but are not necessarily more or less likely to use antibiotics for these conditions compared to children without disabilities. However, gender, country and impairment type disparities persist. Targeted efforts are needed to address these health inequities and ensure equitable access to care.

**Funding:**

This research was partially supported by 10.13039/501100001809National Natural Science Foundation of China (grant number: 72374228, 72074234), 10.13039/501100021171Guangdong Basic and Applied Basic Research Foundation (grant number: 2023A1515010163), Guangzhou Basic and Applied Basic Research Program (grant number: 2025A04J5118), and Fundamental Scientific Research Funds for Central Universities, China (grant number: SYSU-25wkjc02).

**Research in context**

**Evidence before this study**

Child disability is an emerging global health priority. Many of these children face numerous challenges, such as discrimination, inadequate healthcare and support services, which often leads to adverse health and social consequences. The Sustainable Development Goals (SDGs) calls the necessary to pay attention to the potential impact of antimicrobial resistance (AMR) on high-risk groups for infections, especially children with disabilities. Previous evidence showed that children with disability are at a higher risk of childhood illnesses and premature death due to difficulties in accessing healthcare services and poverty. The susceptible to severe illness and delayed or inappropriate treatment greatly elevate the risk of AMR among children with disabilities. We searched PubMed and Google Scholar using the combination “disability” and “antimicrobial resistance”, “antibiotic”, “children”, “multiple indicator cluster survey (MICS)”, and “infection”. We found one letter in the academic literature that suggests incorporating disability considerations into AMR strategy, but this was only a proposal and did not provide quantitative evidence. Furthermore, we found no quantitative studies examining the Antibiotic use patterns of children with disability.

**Added value of this study**

Using the round 6 of the UNICEF-supported multi-country Multiple-Indicator Cluster Surveys (MICS), this study is the first to assess antibiotic use in children living with disability for common illness conditions and compare the difference in children by sex and country. Children with disabilities are at higher risk of developing common illnesses than children without disabilities. Currently, there was no evidence that children with disability had different odds of antibiotic use for common illnesses. However, there are significant differences in reported antibiotic use among children with disability in some countries and specific impairment type compared to children without disability. And we found gender inequities exists: girls with disability are less likely to take antibiotics than others.

**Implications of all the available evidence**

This study is the first attempt to explore the relationship between antibiotic use and childhood disability and provided reliable evidence of the susceptible to common illnesses among children with disability. Our findings once again emphasize the concern for children with disability. Currently, this topic receives insufficient attention and needs future exploration to verify the health inequalities and disability status in developing countries. Addressing the health inequity for children with disabilities is crucial to ensuring children welfare and achieving leaving no one behind. As a vulnerable group, they are not only a high-risk group for common diseases, but also a high-risk group for antibiotic resistance. They face more health risk factors compared to other populations or normal children. In the past, there was a lack of attention to the prevention of antibiotic resistance in children with disabilities. Our quantitative evidence provides some inspiration for antibiotic prevention and treatment strategies for children with disabilities.

## Introduction

Globally, nearly 240 million children are living with disabilities, accounting for roughly one in ten.^1^ This figure is expected to rise due to population growth and poor health outcomes in low- and middle-income countries (LMICs).^2^ Children with disabilities face numerous challenges, including stigma, discrimination, and limited access to adequate healthcare and support services, often leading to adverse health and social consequences.^3^

Child disability is increasingly recognized as a global health priority. The Sustainable Development Goals (SDGs) aim to reduce the burden of child mortality, improve overall child health, and achieve gender equality.^4^ Due to physical vulnerabilities, barriers to accessing healthcare, and the effects of poverty, children with disabilities are at higher risk of premature death and lifelong consequences.^5^ Research has shown that children with disabilities in sub-Saharan Africa have greater odds of experiencing acute respiratory infection (ARI), diarrhea, and fever,^6^ which are leading causes of death and disease among children in LMICs, accounting for nearly one-third of child deaths.^7^

Recent studies have highlighted the need to consider the potential impact of antimicrobial resistance (AMR) on high-risk groups for infections, including individuals with disabilities, which presents a major concern.^8^ On the one hand, they are more susceptible to severe illness due to their vulnerability; on the other hand, socioeconomic factors such as poverty^9^ create barriers to accessing timely and appropriate healthcare. These challenges can result in delayed or inadequate treatment of infections, increasing the risk of AMR in this population.

Understanding the association between disability status and antibiotic use is essential for formulating strategies to address the urgent intersection of AMR and disability. Such efforts can improve child well-being and advance health equity. However, there is currently a lack of research exploring this critical area.

Using data from round six of the UNICEF-supported Multiple Indicator Cluster Surveys (MICS), this study aimed to assess antibiotic use among children with disabilities for common illnesses and to identify associated factors.

## Methods

### Study populations

The data used in this study were extracted from the sixth round of the MICS conducted between 2017 and 2023. This comprehensive survey program was carried out in LMICs to collect nationally and sub-nationally representative data on key health and development indicators.^10^

The MICS uses a cross-sectional, household survey method based on standardized questionnaires administered by trained data collectors, who interview mothers or primary caregivers about their children. The surveys employ a stratified multistage sampling design, based on existing sampling frames. Enumeration areas from national censuses were randomly selected, followed by random sampling of household clusters within these areas.^10^^,^^11^ The MICS data, publicly accessible through UNICEF's online platform, include variables central to this study, such as disability status and antibiotic use for common illnesses.

**Fig. 1:**
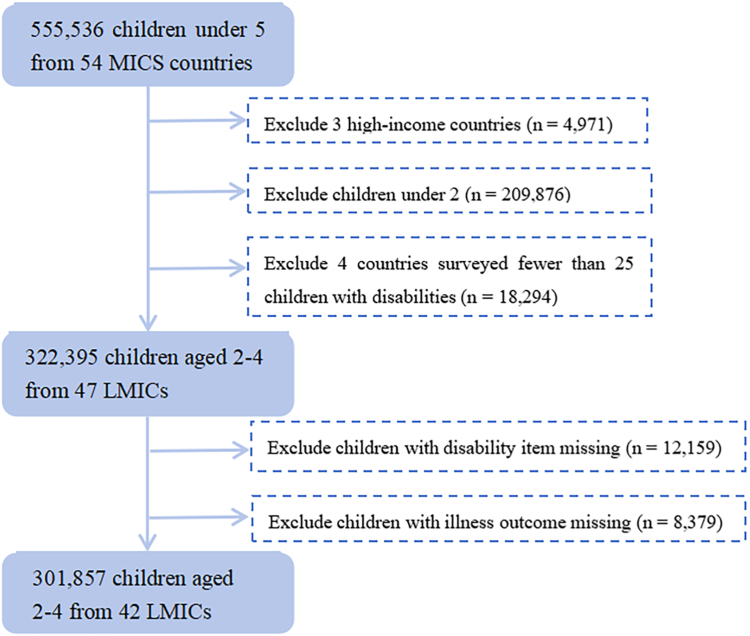
**Flowchart of selection of participants aged 2–4 years from the Multiple Indicator Cluster Survey (MICS) countries**.

We extracted individual participant data for MICS6, including 555,536 children under 5 from 54 countries. The data were publicly accessible on the UNICEF website as of June 2024. We excluded 3 high income countries. And to minimize bias due to limited sample sizes, we excluded 4 countries where fewer than 25 children with disabilities were surveyed, as recommended by UNICEF.^12^ For this reason, MICS6 data of Tuvalu, Montenegro, Jamaica and Thailand are available but not included. All children with complete data on outcome variables and disability status were included in the analysis of this current study. Five countries (Kosovo, Republic of North Macedonia, Serbia, Turkmenistan and Uzbekistan) were excluded due to the complete missing of illness outcome and disability variables (Fig. 1).

The final dataset comprised 301,857 children aged 2–4 years from 42 countries. Table S1 represented a summary of countries and sample sizes. Of these, 17 countries were from sub-Saharan Africa, representing the largest proportion (40.4%). Lower-middle-income countries made up nearly half of the sample, while low-income and upper-middle-income countries contributed similar proportions (Table S2).

### Data collection and measurements

#### Disability

The MICS6 surveys incorporated the Child Functioning Module (CFM) validated by UNICEF and the Washington Group on Disability Statistics to identify children with disabilities aged 2–17 years.^13^ We focused on children aged 2–4 whose common illnesses and use of antibiotics were reported.

Disability status was assessed across eight functional domains: seeing, hearing, walking, controlling behaviour, learning, playing, communication, and fine motor skills.^14^ A child was classified as living with disability if their caregiver reported “a lot of difficulty” (or “a lot more” for the variable on controlling behaviour) or “cannot do at all” in one or more domains, based on validated UNICEF cut-off.^15^ Children were considered without disability if they were reported to have “no difficulty” or “some difficulty” across all domains. Those with missing data on all CFM questions were excluded from the analysis. In total, 12,159 (3.8%) cases were removed. The percentage of disability status in each country is shown in Figure S1. We omitted the individuals with missing values instead of any imputation.

#### Common illness

Data on three common illnesses—diarrhea, fever, and ARI—were collected. Diarrhea and fever were identified based on caregiver-reported symptoms within the two weeks preceding the survey interview. ARI was defined as an illness involving rapid or difficult breathing during the same period, where the symptoms were perceived by caregivers to stem from chest problems or a blocked/runny nose.^16^

#### Antibiotic use

For each of the three illnesses (ARI, diarrhea, and fever), caregivers were asked whether antibiotics were used to treat the condition.

#### Covariates

Covariates included child age, sex, place of residence (urban/rural), mother's highest education level, the number of children under 5 in the household, and country.^17^ Child age and sex were caregiver-reported, while residence location was determined by interviewers based on cluster designations provided by UNICEF or national statistical agencies.

### Statistical analysis

Participant characteristics were described using means (standard deviations) for continuous variables and numbers (proportions) for categorical variables.

Weighted logistic regression was used to analyze the association between disability status (yes/no) and antibiotic use (yes/no) for each illness (diarrhea, fever, and ARI), controlling for country, age, sex, residence place, mother's education and number of children under 5. Each household had a defined selection probability, enabling national estimates using sample weights directly available in MICS.^10^ Results were reported as unadjusted and adjusted odds ratios (ORs) with 95% confidence intervals (CIs).

To study the relationship between children living with disabilities and antibiotic use in sex, nation and disability subtypes subgroups, similar analysis was conducted and the same weighted logistic regression model was used.

We performed sensitivity analyses, addressing the issue of missing values through 20 multiple imputations using chained equations.^18^

All analyses were conducted using Stata, version 17, while graphs were generated in R, version 4.4.1. Statistical significance was set at *p* < 0.05.

STROBE Statement–Checklist of items that should be included in reports of cross-sectional studies, was provided in this study.

### Ethics statement

The data are publicly accessible and contain no personally identifiable information. Therefore, this analysis does not require ethics approval.

### Role of funding source

The funders played no role in the design, collection, analysis, or interpretation of results.

## Results

There were 301,857 children aged 2–4 years included in the study. Table 1 presents baseline characteristics by disability status. The overall prevalence of disability was 6.9% (n = 20,819). Among the reported difficulties, problems with controlling behaviour (36.3%) and learning (35.9%) were the most common, while hearing difficulties (7.0%) were the least reported. The distributions of basic characteristics of our study participants were similar to those 2–4 years included in the MICS survey (Table S3).

**Table 1:** Characteristics by disability status of 301,857 children aged 2–4 years and their families from 42 Multiple Indicator Cluster Survey (MICS) countries**.**^b^

Characteristics	All children (N = 301,857)			Children with disabilities (n = 20,819)		
	Without disabilities (n = 281,038)	With disabilities (n = 20,819)	*p*-value	Girls (n = 9371)	Boys (n = 11,448)	*p*-value
Male (n, %)	142,634 (50.7)	11,448 (55.0)	**<0.001**	–	–	
Age (n, %)			**<0.001**			0.223
2 years	88,695 (31.5)	8512 (40.9)		3875 (41.4)	4637 (40.5)	
3 years	96,876 (34.5)	6531 (31.4)		2950 (31.5)	3581 (31.3)	
4 years	95,467 (34.0)	5776 (27.7)		2546 (27.1)	3230 (28.2)	
Rural residence (n, %)	192,186 (68.4)	15,617 (75.0)	**<0.001**	2289 (24.4)	2913 (25.5)	0.091
Mother's highest level of education (n, %)			**<0.001**			**<0.001**
No education	113,399 (41.0)	11,406 (55.0)		5301 (56.8)	6105 (53.5)	
Primary	60,934 (22.0)	4307 (20.8)		1872 (20.1)	2435 (21.4)	
Secondary	46,981 (17.0)	2657 (12.8)		1151 (12.3)	1506 (13.2)	
Higher	55,497 (20.0)	2364 (11.4)		1005 (10.8)	1359 (11.9)	
Number of children under 5 in the household (n, %)			**<0.001**			**0.005**
One	116,813 (41.6)	7243 (34.8)		3151 (33.6)	4092 (35.7)	
Two	104,301 (37.1)	8039 (38.6)		3625 (38.7)	4414 (38.5)	
Three	37,226 (13.2)	3289 (15.8)		1551 (16.6)	1738 (15.2)	
Four	12,440 (4.4)	1173 (5.6)		547 (5.8)	626 (5.5)	
Five or more	10,258 (3.7)	1075 (5.2)		497 (5.3)	578 (5.1)	
Reported disabilities^a^						
Seeing	–	2248 (10.8)		1044 (11.2)	1204 (10.5)	0.143
Hearing	–	1452 (7.0)		691 (7.4)	761 (6.7)	**0.04**
Walking	–	3176 (15.3)		1461 (15.6)	1715 (15.0)	0.215
Controlling Behaviour	–	6154 (36.3)		2527 (33.5)	3627 (38.5)	**<0.001**
Playing	–	3101 (14.9)		1437 (15.3)	1664 (14.5)	0.108
Learning	–	7465 (35.9)		3546 (37.9)	3919 (34.3)	**<0.001**
Communication	–	6550 (31.5)		2953 (31.5)	3597 (31.4)	0.883
Fine Motor Skills	–	2357 (11.3)		1116 (11.9)	1241 (10.9)	**0.015**

a The percentages do not add up to 100% as children could have multiple disabilities or present with overlapping difficulties.

b Bold values indicate *p* < 0.05

Children with disabilities were more likely than those without disabilities to be male (55.0% vs. 50.7%, *p* < 0.001), younger (*p* < 0.001), reside in rural areas (75.0% vs. 68.4%, *p* < 0.001), and have mothers with lower levels of education (*p* < 0.001).

Among children with disabilities, girls were more likely than boys to have mothers with lower levels of education (*p* < 0.001) and to experience difficulties with hearing (*p* = 0.04), learning (*p* < 0.001), and fine motor skills (*p* = 0.015). However, they were less likely than boys to exhibit difficulties with controlling behaviour (*p* < 0.001).

Table 2 shows that the two-week prevalence of ARI (20.7% vs. 11.9%, *p* < 0.001), diarrhea (22.8% vs. 13.3%, *p* < 0.001), and fever (32.5% vs. 23.5%, *p* < 0.001) was significantly higher among children with disabilities compared to those without disabilities. These findings were confirmed through adjusted analyses, which showed higher odds of these common illnesses overall and by gender, except for diarrhea (Table 2). Figures S2–S4 shows the adjusted odds ratios for having ARI, diarrhea and fever disaggregated by country according to disability status.

**Table 2:** Adjusted odds ratios for Acute Respiratory Infection (ARI), diarrhea, and fever in children (2–4 years) with disabilities compared to those without disabilities.^c^

	Children without disabilities, N (%)	Children with disabilities, N (%)	*p*-value	Overall aOR^a^	Girls with disabilities aOR^b^	Boys with disabilities aOR^b^
ARI	33,007 (11.9)	4227 (20.7)	**<0.001**	**1.78 [1.34, 2.36]**	**1.67 [1.22, 2.30]**	**1.86 [1.25, 2.76]**
Diarrhea	37,327 (13.3)	4708 (22.8)	**<0.001**	1.16 [0.96, 1.40]	1.28 [0.97, 1.69]	1.08 [0.84, 1.39]
Fever	65,877 (23.5)	6697 (32.5)	**<0.001**	**1.54 [1.22, 1.96]**	**1.37 [1.05, 1.81]**	**1.66 [1.19, 2.33]**

a Adjusted for country, age, sex, residence place, mother's education and number of children under 5.

b Adjusted for country, age, residence place, mother's education and number of children under 5.

c Bold values indicate *p* < 0.05.

Furthermore, the odds of having an illness did not differ between girls with disabilities and boys with disabilities (Appendix Table S4).

The rate of reported antibiotic use among children with disabilities experiencing ARI, diarrhea, and fever was 40.6%, 10.1%, and 32.6%, respectively. There was no significant difference in reported antibiotic use for ARI (aOR = 1.13, 95% CI: 0.68–1.87), diarrhea (aOR = 0.93, 95% CI: 0.64–1.36), or fever (aOR = 1.23, 95% CI: 0.81–1.86) between children with and without disabilities (Table 3).

**Table 3:** Adjusted odds ratios for reported antibiotic use for Acute Respiratory Infection (ARI), diarrhea, and fever in children (2–4 years) with disabilities compared to those without disabilities.^c^

	Children without disabilities, N (%)	Children with disabilities, N (%)	*p*-value	Overall aOR^a^	Girls with disabilities aOR^b^	Boys with disabilities aOR^b^
ARI	11,875 (43.1)	1538 (40.6)	**0.003**	1.13 [0.68, 1.87]	0.66 [0.38, 1.15]	1.38 [0.69, 2.75]
Diarrhea	3998 (12.1)	444 (10.1)	**<0.001**	0.93 [0.64, 1.36]	**0.78 [0.63, 0.96]**	1.02 [0.57, 1.82]
Fever	20,069 (32.5)	2116 (32.6)	0.809	1.23 [0.81, 1.86]	0.78 [0.49, 1.24]	1.48 [0.85, 2.58]

a Adjusted for country, age, sex, residence place, mother's education and number of children under 5.

b Adjusted for country, age, residence place, mother's education and number of children under 5.

c Bold values indicate *p* < 0.05.

However, girls with disabilities reported significantly lower antibiotic use for diarrhea compared to girls without disabilities (aOR = 0.78, 95% CI: 0.63–0.96). Additionally, girls with disabilities had lower odds of reported antibiotic use for ARI compared to boys with disabilities (Table S4; aOR = 0.53, 95% CI: 0.29–0.98).

**Fig. 2:**
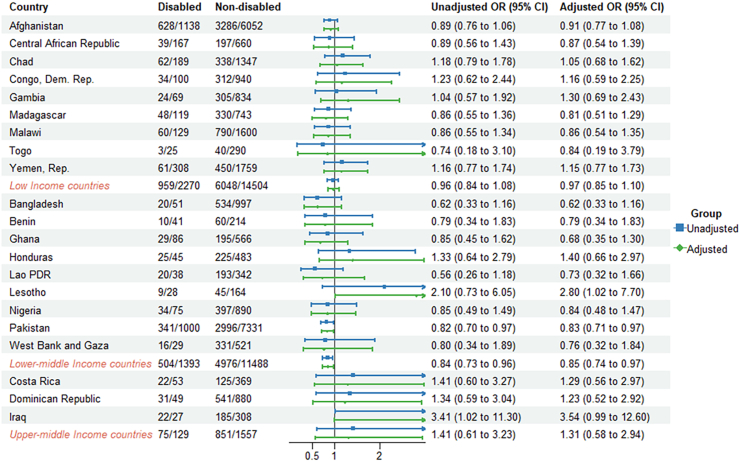
**Odds ratios (ORs) for reported antibiotic use for ARI in children (2–4 years) with disabilities compared to those without disabilities**.

**Fig. 3:**
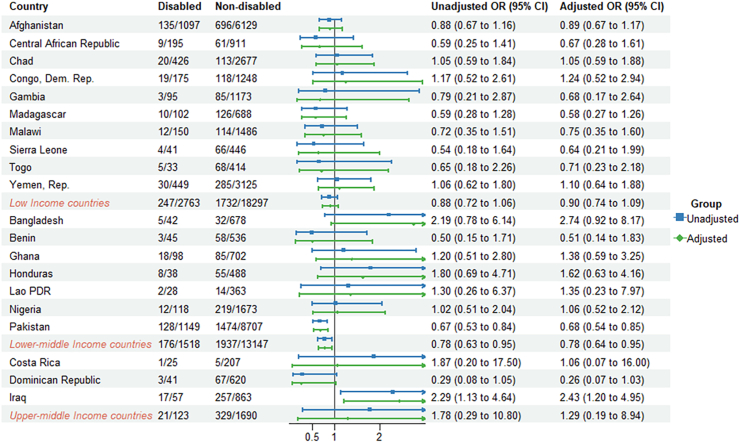
**Odds ratios (ORs) for reported antibiotic use for diarrhea in children (2–4 years) with disabilities compared to those without disabilities**.

**Fig. 4:**
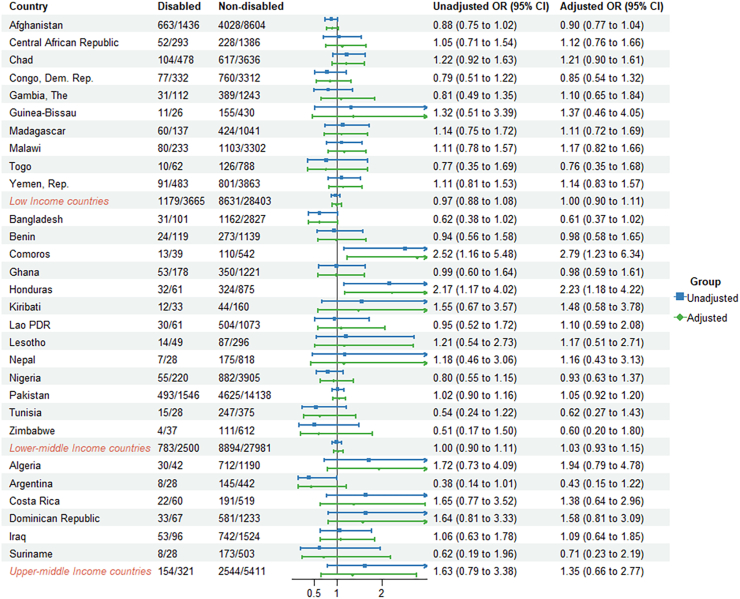
**Odds ratios (ORs) for reported antibiotic use for fever in children (2–4 years) with disabilities compared to those without disabilities**.

Comparing the countries' economic status, the lower-middle income countries had lower odds of reported antibiotic use for ARI and diarrhea (aOR = 0.85, 95% CI: 0.74–0.97, aOR = 0.78, 95% CI: 0.64–0.95, respectively) compared to children without disabilities (Fig. 2, Fig. 3, Fig. 4, Table S5).

Reported antibiotic use for children with disabilities varied across countries. Within countries, the proportion of reported antibiotic use for children with disability with ARI ranged from 9.2% in Togo to 85.3% in Iraq. For diarrhea and fever, the proportion ranged from 3.6% in Central African Republic to 48.6% in Iraq and from 10.2% in Zimbabwe to 70.8% in Algeria (Figures S5–S7). Reported antibiotic use for children with disabilities varied across countries. In Lesotho, children with disabilities had higher odds of reported antibiotic use for ARI (aOR = 2.80, 95% CI: 1.02–7.70) compared to children without disabilities. In Iraq, children with disabilities were significantly more likely to use antibiotics for diarrhea, with an adjusted odds ratio of 2.43 (95% CI: 1.20–4.95). Conversely, in Pakistan, children with disabilities had lower odds of antibiotic use for ARI (aOR = 0.83, 95% CI: 0.71–0.97) and diarrhea (aOR = 0.68, 95% CI: 0.54–0.85) compared to children without disabilities. For antibiotics used to treat fever, children with disabilities in Comoros (aOR = 2.79, 95% CI: 1.23–6.34) and Honduras (aOR = 2.23, 95% CI: 1.18–4.22) had higher odds of antibiotic use compared to their counterparts without disability (Fig. 2, Fig. 3, Fig. 4).

There were also differences in reported antibiotic use between children with different types of disabilities and their counterparts without disabilities. Children with seeing difficulties had a lower odds of reported antibiotic use for ARI (aOR = 0.65, 95% CI: 0.47–0.89) (Table 4). For diarrhea, children with problems controlling behaviour (aOR = 0.66, 95% CI: 0.48–0.91) and learning difficulties (aOR = 0.76, 95% CI: 0.60–0.96) were less likely to report antibiotic use. No significant difference was observed in reported antibiotic use for fever among children with different disabilities compared to their peers without disabilities.

**Table 4:** Adjusted odds ratios (aORs) for reported antibiotic use in children (2–4 years) with different types of disabilities compared to those without disabilities**.**^b^

	Reported antibiotic use for ARI		Reported antibiotic use for diarrhea		Reported antibiotic use for fever	
	N (%)	aOR [95% CI]^a^	N (%)	aOR [95% CI]^a^	N (%)	aOR [95% CI]^a^
Children without disabilities	11,875 (43.1)	/	3998 (12.1)	/	20,166 (32.5)	/
Reported disabilities						
Seeing	105 (27.5)	**0.65 [0.47, 0.89]**	46 (10.0)	0.75 [0.48, 1.18]	163 (29.7)	1.09 [0.84, 1.42]
Hearing	101 (35.6)	0.87 [0.62, 1.22]	30 (8.8)	0.79 [0.49, 1.27]	135 (28.8)	0.79 [0.51, 1.20]
Walking	276 (41.5)	2.12 [0.71, 6.31]	94 (11.5)	0.89 [0.65, 1.22]	384 (32.5)	1.41 [0.58, 3.40]
Controlling behavior	274 (34.9)	0.57 [0.32, 1.00]	93 (10.6)	**0.66 [0.48, 0.91]**	466 (29.9)	1.61 [0.81, 3.16]
Playing	327 (47.7)	1.25 [0.68, 2.33]	107 (12.8)	1.10 [0.83, 1.46]	429 (37.0)	1.38 [0.94, 2.02]
Learning	722 (47.4)	0.87 [0.47, 1.62]	167 (9.2)	**0.76 [0.60, 0.96]**	905 (35.2)	1.23 [0.66, 2.30]
Communication	535 (40.4)	1.42 [0.66, 3.03]	151 (9.1)	1.05 [0.47, 2.34]	734 (32.2)	0.99 [0.52, 1.90]
Fine motor skills	237 (46.6)	0.86 [0.54, 1.35]	78 (13.0)	0.99 [0.71, 1.38]	293 (33.7)	0.89 [0.66, 1.20]

a Adjusted for country, age, sex, residence place, mother's education and number of children under 5.

b Bold values indicate *p* < 0.05.

The sensitivity analyses confirmed consistency of the above-reported results (Appendix 5).

## Discussion

In this study of 301,857 children aged 2–4 years from 42 LMICs, the odds of having ARI and fever in the past two weeks were higher in children with disabilities than in children without disabilities, and the absolute differences were substantial. There was no evidence that children with disabilities had different odds of reported antibiotic use for ARI, diarrhea, and fever. However, significant differences existed across countries. The lower-middle income countries had lower odds of reported antibiotic use for ARI and diarrhea. In Lesotho, Iraq, Comoros and Honduras, children with disabilities had higher odds of reported antibiotic use, compared to lower odds of reported antibiotic use in Pakistan. Additionally, children with seeing, controlling behaviour, or learning impairments had lower reported antibiotic use compared to children without disabilities. The study further found that girls with disabilities were less likely to use antibiotics compared to their peers without disabilities and boys with disabilities.

We provided reliable evidence that children with disabilities are more susceptible to certain common childhood illnesses, which is consistent with the findings of previous studies. A report from UNICEF indicates a higher incidence of ARI among children with disabilities (approximately 53%).^1^ Rotenberg found that children with disabilities from countries in sub-Saharan Africa were more likely to experience ARI, diarrhea, and fever based on MICS data.^6^ Possible explanations are, firstly, children with disabilities tend to have worse health status, which may increase their vulnerability to infections.^19^^,^^20^ Secondly, disability and poverty are believed to operate in a cycle.^21^ Children with disabilities living in poverty may be more susceptible to poor sanitary conditions and limited access to clean drinking water and sanitation facilities.^22^^,^^23^ These factors are significant contributors to infectious diseases and likely contributed to the higher prevalence of common illnesses observed in this study. Thirdly, children with disabilities may face barriers in accessing healthcare. Although small absolute differences were found in access to care between children with and without disabilities, children with disabilities are at a disadvantage in real life when it comes to healthcare services due to inequitable conditions in all facets of life, such as higher medical costs and insufficient insurance coverage.^24^ Our study did not find any gender differences in the occurrence of illnesses between boys and girls with disabilities. Overall, the substantial absolute differences in illness rates between children with and without disabilities highlight an urgent need for targeted interventions to reduce health inequalities for children with disabilities.

A strong association between disability status and reported antibiotic use was not observed in our study. When children with disabilities had ARI or diarrhea, reported antibiotic use appeared to be lower than in children without disabilities, although the difference was relatively small. Our empirical results indicated no difference in odds of antibiotic use between children with and without disabilities. In fact, few studies have explored the relationship between disability status and antibiotic use prior to our study. Therefore, this study is a groundbreaking exploration of antibiotic use in children with disabilities. Another study indirectly supports our findings, as it found no correlation between children with disabilities and the likelihood of seeking care.^6^

It is worth noting that our analysis was not able to distinguish between justified and unjustified use of antibiotics. Antibiotic use is only recommended in a small subset of common childhood illnesses.^25^ Based on the current data of MICS, we are unable to distinguish whether antibiotics are used rationally. Our study aimed to addresses the critical gap in understanding antibiotic exposure patterns among children with disabilities. We highlight the need of further research on this area and collecting more data to examine the possible inequalities in the rational use of antibiotics.

The study also highlighted global differences. In our study, we found variations in antibiotic use across the three common illness conditions, despite a lack of overall differences between the children with and without disabilities. However, country differences cannot be ignored. The lower-middle income countries had lower odds of reported antibiotic use for ARI and diarrhea. Compared to children without disabilities, those with disabilities had higher odds of reported antibiotic use in Lesotho, Iraq, Comoros, and Honduras, but lower odds of reported antibiotic use in Pakistan. These variations may be attributable to a wide range of factors, such as differences in individual and family characteristics, healthcare systems, and social welfare programs.^26^ It is important to acknowledge the unique challenges each country faces.^27^ Unfortunately, we are unable to dissect the specific reasons for these country differences due to the limitation of the existing data. But the disparities suggest that local contexts should be considered, and targeted interventions should be implemented based on the specific needs and challenges faced by children with disabilities in different countries.

The association between antibiotic use and disability status varies by the types of disabilities, according to the findings of our study. Children with problems in seeing, controlling behaviour and learning reported lower odds of antibiotic use for common illnesses compared to those without disabilities, possible explanations include high levels of stigma, physical and emotional exhaustion with social exclusion of the child and caregiver, which may resulting in reduced opportunities to seek care and get treatment.^28^ Understanding the underlying reasons behind these complex association between antibiotic use and disability status requests further studies. But this discovery indicating that in addition to focusing on the overall status of children with disabilities, particular attention should also be given to children with specific types of disabilities. Targeted interventions and support for specific subgroup should also be highlighted.

It is noteworthy that we found lower odds of reported antibiotic use when comparing girls with disabilities both to girls without disabilities and boys with disabilities. Gender-related social norms often disadvantage girls in terms of access to healthcare services.^29^ For girls with disabilities, this inequality may be even more pronounced.^30^ Although our results cannot distinguish whether there is underuse or overuse of antibiotics, or whether the use of antibiotics is appropriate,^31^ millions of people in LMICs still lack access to antibiotics and other life-saving medicines.^32^ Compared to the antibiotic use rate for children under five in previous studies,^33^ the rate of antibiotic use among children with disabilities in our results is lower. Our study provides evidence that greater attention should be given to children with disabilities, especially girls with disabilities, as these children in LMICs may still face challenges in accessing medication.^34^

This study provides a novel analysis of key health indicators for children with disabilities within the MICS. The health of children with disabilities is of great significance for achieving the SDGs. However, there is a significant lack of monitoring, evaluation, and data related to children with disabilities. Our study offers crucial evidence regarding the prevalence of common illnesses among children with disabilities, highlighting the substantial differences between children with and without disabilities in this context. Additionally, we are the first to examine the relationship between children with disabilities and antibiotic use, which helps fill a gap in this area of research and represents a key contribution. Overall, our study emphasizes the need for greater attention and resources to narrow the health gap between children with and without disabilities in LMICs, ultimately improving the well-being and health of children with disabilities.

This study has limitations that need to be considered when interpreting the results. First, due to data constraints, we only included children aged 2–4 years, and the sample may not adequately represent all children with disabilities. Due to social and cultural reasons, there may existed reporting biases about disabilities across different countries. We have included “country” as a covariate in the statistical modelling, we also adjusted for socioeconomic variations in the modelling to further mitigate the risk of contextual biases. Additionally, disability information was collected using the CFM tool, which differs from the traditional understanding of physical disabilities and lacks specific examples of the types. So, caution should be taken when interpreting specific types of disabilities. Furthermore, the wide confidence intervals of the results for some countries require cautious interpretations. Our results are also subject to potential recall bias, as the primary outcomes were self-reported by caregivers. However, previous studies have found high levels of agreement between medical records and caregiver-reported antibiotic use in children in LMICs.^35^ Finally, readers need to take precautions in interpreting the multiple comparison results as they were not corrected to reduce type I errors. The observed associations should be interpreted as preliminary evidence to guide targeted future studies.

In conclusion, this study provides evidence of a higher prevalence of common childhood illnesses among children with disabilities across 42 LMICs globally. Although, overall, there is no evidence indicating a difference in reported antibiotic use for these common illness conditions, we found that attention should be focused on sex differences, particularly among girls, as well as national-level variations in antibiotic use. A significant health gap remains between children with and without disabilities. Currently, this topic receives insufficient attention and needs future exploration to verify the health inequalities and disability status in developing countries. Addressing health inequity for children with disabilities is crucial to ensuring the welfare of children with disabilities and achieving “leaving no one behind”.^4^

## Contributors

LPY, SQC, SYQ and MLX designed the study. SYQ and MLX conducted the analysis and wrote and edited the manuscript. YYW, CJL, XYL, XYY, HHX, RNW, ZXM, FQM, XPZ, GL, HK, SQC and LPY have substantial contributions to data materials, interpretation of data and results. YYW, CJL, LPY and SQC revised it critically for important intellectual content. LPY and SQC supervised this work. LPY, SQC and SYQ have accessed and verified the underlying data. LPY, SQC, SYQ and MLX were responsible for the decision to submit the manuscript, and all authors contributed to final approval of the paper.

## Data sharing statement

The datasets supporting this research were sourced from publicly available repositories. Child-level data can be retrieved through UNICEF's Multiple Indicator Cluster Surveys (MICS) (https://mics.unicef.org/surveys).

## Declaration of interests

All authors declared no competing interests.

## Appendix A Supplementary data

Translated Abstracts
