# Supplementary material for: Antibiotic use for common illnesses in children living with disability: a multi-country study across 42 low- and middle-income countries
Source: eClinicalMedicine. 2025 Jul 2;85:103326. doi: 10.1016/j.eclinm.2025.103326 (PMC12270707; doi:10.1016/j.eclinm.2025.103326)
Supplement: Translated Abstracts [file mmc1.docx]

# The following translations in Chinese were submitted by the authors and we reproduce them as supplied. They have not been peer reviewed. Our editorial processes have only been applied to the original abstract in English, which should serve as reference for this manuscript

# Antibiotic use for common illnesses in children living with disability: a multi-country study across 42 low- and middle-income countries

# 儿童残疾状况与常见疾病抗生素使用情况：一项包含42个低收入和中等收入国家的多国研究

邱声越^a, #^, 徐茗丽^b, #^, 吴远洋^c^, 刘朝杰^d^, 李溪影^a^, 杨欣怡^a^, 夏浩海^a^, 王若楠^a^, 马子舒^a^, 孟凡芊^a^, 张新平^c^, 刘国恩^e,f^, Hannah Kuper ^g^, 陈山泉^g,*^, 杨廉平^a,f,h,*^

^a^ 中山大学公共卫生学院，中国广州

^b^ 华南师范大学政治与公共管理学院，中国广州

^c^ 华中科技大学同济医学院医药卫生管理学院，中国武汉

^d^ 澳大利亚乐卓博大学心理与公共卫生学院，澳大利亚墨尔本

^e^ 北京大学国家发展研究院，中国北京

^f^ 北京大学全球健康发展研究院，中国北京

^g^ 伦敦卫生与热带医学院流行病学与人口健康学院国际残疾证据中心，英国伦敦

^h^ 中山大学国家治理研究院全球卫生研究中心，中国广州

^#^这些作者对这项工作贡献均等，为共同第一作者

^*^通讯作者：杨廉平教授，中山大学公共卫生学院，中国广州越秀区中山二路74号，510080. 电子邮箱：yanglp7@mail.sysu.edu.cn |电话：+86-02087331470

陈山泉教授，伦敦卫生与热带医学院流行病学与人口健康学院国际残疾证据中心

电子邮箱：shanquan.chen@lshtm.ac.uk

**摘要**

**研究背景**

联合国儿童基金会报告显示，全球残疾儿童约有2.4亿人。探究残疾状况与儿童常见疾病抗生素使用之间的关联，有助于制定应对抗生素耐药性（antimicrobial resistance, AMR）与残疾这一关键交叉问题的策略。

**研究方法**

本研究数据来自于联合国儿童基金会支持的的多指标类集调查(2017–2023)（Multiple Indicator Cluster Surveys，MICS）的横断面研究，覆盖42个低收入和中等收入国家。残疾状况采用华盛顿残疾统计小组开发的儿童功能模块（Child Functioning Module，CFM）评估。通过照护者报告采集儿童因常见疾病使用抗生素的情况。构建Logistic回归模型，在控制年龄、性别、居住地、母亲教育水平、家庭5岁以下儿童数量及国家因素后，分析儿童残疾状况与两周内急性呼吸道感染（acute respiratory infection, ARI）、腹泻和发热的患病情况，以及针对这些疾病抗生素使用之间的关联。

**研究发现**

研究纳入301,857名儿童，其中6.9%患有残疾。与非残疾儿童相比，残疾儿童罹患急性呼吸道感染（aOR = 1.78，95% CI 1.34–2.36）和发热（aOR = 1.54 95% CI 1.22–1.96）的可能性更高。残疾儿童与非残疾儿童在罹患急性呼吸道感染（aOR = 1.13，95% CI 0.68–1.87）、腹泻（aOR = 0.93，95% CI 0.64–1.36）、发热（aOR = 1.23，95% CI 0.81–1.86）时的抗生素使用无显著差异。但各国情况存在差异：中等偏下收入国家儿童因急性呼吸道感染（aOR = 0.85，95% CI 0.74–0.97）和腹泻（aOR = 0.78，95% CI 0.64–0.95）使用抗生素的几率较低。莱索托、伊拉克、科摩罗和洪都拉斯四国的残疾儿童抗生素使用几率较高，而巴基斯坦残疾儿童的抗生素使用几率较低。亚组分析显示，残疾女孩较非残疾女孩因腹泻使用抗生素的可能性更低（aOR = 0.78, 95% CI 0.63–0.96），残疾女孩较残疾男孩使用抗生素的几率也更低（aOR = 0.53, 95% CI 0.29–0.98）。此外，存在视力障碍、行为控制障碍或学习障碍的儿童使用抗生素的几率相较非残疾儿童也更低。

**研究结论**

残疾儿童罹患常见疾病的风险更高，但因这些疾病使用抗生素的几率与非残疾儿童相比无显著差异。然而，不同性别、国家及残疾类型的残疾儿童与非残疾儿童相比，抗生素用药差异仍然存在。需要采取针对性措施以解决这些健康不平等问题，确保医疗服务的公平可及。

**基金资助**

这项研究得到了中国国家自然科学基金（72374228，72074234）和中国广东省自然科学基金（2023A1515010163)的资助。

**关键词：**儿童疾病；残疾；抗生素；公平；中低收入国家
