# Supplementary material for: Antibiotic use for common illnesses in children living with disability: a multi-country study across 42 low- and middle-income countries
Source: eClinicalMedicine. 2025 Jul 2;85:103326. doi: 10.1016/j.eclinm.2025.103326 (PMC12270707; doi:10.1016/j.eclinm.2025.103326)
Supplement: Supplementary Material [file mmc2.docx]

**Supplementary Materials**

**Appendix 1**

**Table S1. Summary of countries, survey year and number of children in study sample**

| **Country** | **Survey year** | **Sample** | **Reported disabilities** |
| --- | --- | --- | --- |
| Afghanistan | 2022-23 | 20,373 | 2,874 (14.1) |
| Algeria | 2018-19 | 9,086 | 234 (2.6) |
| Argentina | 2019-20 | 3,994 | 148 (3.7) |
| Bangladesh | 2019 | 14,056 | 373 (2.7) |
| Belarus | 2019 | 2,274 | 32 (1.4) |
| Benin | 2021-22 | 7,915 | 633 (8.0) |
| Central_African_Republic | 2018-19 | 5,437 | 784 (14.4) |
| Chad | 2019 | 14,153 | 1,524 (10.8) |
| Comoros | 2022 | 2,708 | 172 (6.4) |
| Costa_Rica | 2018 | 2,306 | 157 (6.8) |
| Cuba | 2019 | 3,354 | 68 (2.0) |
| DRCongo | 2017-18 | 12,755 | 943 (7.4) |
| Dominican_Republic | 2019 | 5,082 | 201 (4.0) |
| Eswatini | 2021-22 | 1,376 | 106 (7.7) |
| Fiji | 2021 | 1,266 | 37 (2.9) |
| Gambia | 2018 | 6,169 | 417 (6.8) |
| Georgia | 2018 | 1,606 | 42 (2.6) |
| Ghana | 2017-18 | 5,413 | 551 (10.2) |
| Guinea_Bissau | 2018-19 | 4,601 | 188 (4.09) |
| Honduras | 2019 | 5,177 | 282 (5.5) |
| Iraq | 2018 | 10,179 | 342 (3.4) |
| Kiribati | 2018-19 | 1,270 | 156 (12.3) |
| Kyrgyz_Republic | 2018、2023 | 2,167 | 38 (1.8) |
| Lao | 2017、2023 | 7,206 | 220 (3.1) |
| Lesotho | 2018 | 2,041 | 171 (8.4) |
| Madagascar | 2018 | 7,625 | 750 (9.8) |
| Malawi | 2019-20 | 9,148 | 491 (5.4) |
| Mongolia | 2018 | 3,805 | 89 (2.3) |
| Nepal | 2019 | 4,196 | 78 (1.9) |
| Nigeria | 2021 | 16,884 | 753 (4.5) |
| Pakistan | 2017-20 | 67,580 | 5,564 (8.2） |
| Samoa | 2019-20 | 1,579 | 116 (7.4) |
| Sao_Tome_and_Principe | 2019 | 1,163 | 70 (6.0) |
| Sierra_Leone | 2017 | 7,104 | 514 (7.2) |
| State_of_Palestine | 2019-20 | 3,706 | 88 (2.4) |
| Suriname | 2018 | 2,715 | 93 (3.4) |
| Togo | 2017 | 2,981 | 226 (7.6) |
| Tonga | 2019 | 861 | 63 (7.3) |
| Tunisia | 2018、2023 | 2,172 | 79 (3.6) |
| Viet_Nam | 2020-21 | 2,720 | 39 (1.4) |
| Yemen | 2022-23 | 11,893 | 964 (8.1) |
| Zimbabwe | 2019 | 3,761 | 149 (4.0) |
| **Total** |  | **301,857** | **20,819 (6.9)** |

**
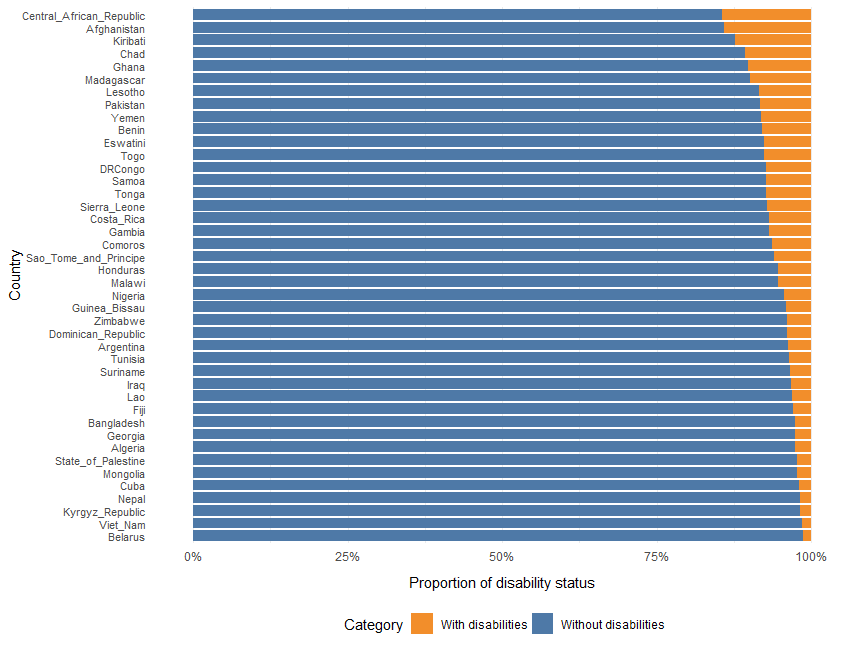
**

**Figure S1: The proportion of children aged 2-4 years with/without disabilities in 42 Multiple Indicator Cluster Survey (MICS) countries.**

**Table S2: Characteristics of 42 Included LMICs from Multiple Indicator Cluster Survey (MICS)**

|  | Countries (n, %) |
| --- | --- |
| Continent |  |
| Sub-Saharan Africa | 17 (40.4) |
| East Asia & Pacific | 7 (16.6) |
| Latin America & Caribbean | 6 (14.2) |
| Middle East & North Africa | 5 (11.9) |
| South Asia | 4 (9.5) |
| Europe & Central Asia | 3 (7.1) |
| Country economic status |  |
| Low Income | 11 (26.1) |
| Lower middle income | 19 (45.2) |
| Upper middle income | 12 (28.5) |

**Table S3: Main characteristics of children before (N = 322,395) and after excluding missing values of disability and health outcomes (N = 301,857)**

| Characteristic | All children aged 2-4  (n = 322,395) | Children included in the  current analysis  (n = 301,857) |
| --- | --- | --- |
| Male (n, %) | 164,576 (51.1) | 154,082 (51.0) |
| Age (n, %) |  |  |
| 2 years | 104,186 (32.3) | 97,207 (32.2) |
| 3 years | 110,274 (34.2) | 103,407 (34.3) |
| 4 years | 107,935 (33.5) | 101,243 (33.5) |
| Rural residence (n, %) | 218,773 (67.9) | 207,803 (68.8) |
| Mother’s highest level of education (n, %) |  |  |
| No education | 128,037 (40.3) | 124,805 (41.9) |
| Primary | 70,469 (22.2) | 65,241 (21.9) |
| Secondary | 53,589 (16.8) | 49,638 (16.7) |
| Higher | 65,906 (20.7) | 57,861 (19.5) |
| Number of children under 5 in the household (n, %) |  |  |
| One | 133,427 (41.4) | 124,056 (41.1) |
| Two | 119,931 (37.2) | 112,340 (37.2) |
| Three | 42,789 (13.3) | 40,515 (13.4) |
| Four | 14,327 (4.4) | 13,613 (4.5) |
| Five or more | 11,921 (3.7) | 11,333 (3.8) |

**Appendix 2**

**Table S4: Adjusted odds ratios of reported antibiotic use for Acute Respiratory Infection (ARI), diarrhoea, and fever in children for boys and girls with disabilities aged 2-4years**

| Variable | Sample size | Boys with disability | Girls with disability |
| --- | --- | --- | --- |
| Have ARI | 20,410 | Reference | 0.83 [0.53,1.29] |
| Reported antibiotic use for ARI | 3,792 | Reference | 0.53 [0.27, 1.02] |
| Have Diarrhoea | 20,628 | Reference | 0.99 [0.72,1.38] |
| Reported antibiotic use for diarrhoea | 4,404 | Reference | 0.66 [0.34, 1.27] |
| Have Fever | 20,593 | Reference | 0.83 [0.57,1.21] |
| Reported antibiotic use for fever | 6,490 | Reference | **0.53 [0.29, 0.98]** |

**Appendix 3**


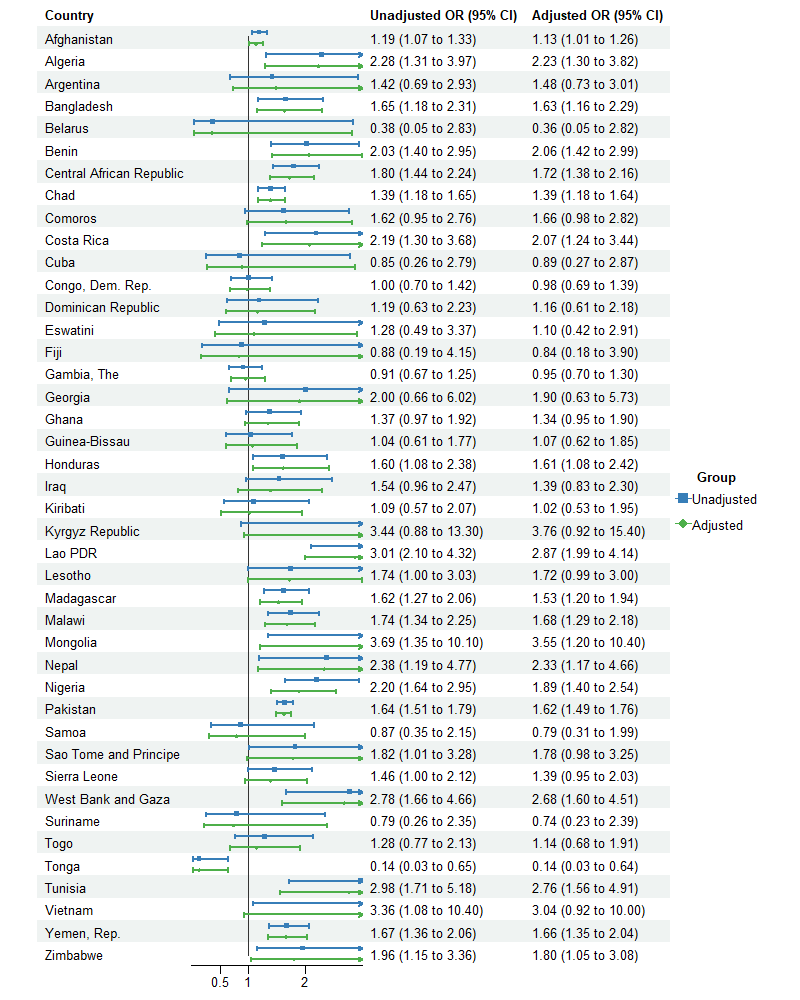


**Figure S2: Adjusted odds ratios for having ARI disaggregated by country according to disability status in children aged 2–4 years from 42 Multiple Indicator Cluster Survey (MICS) countries**


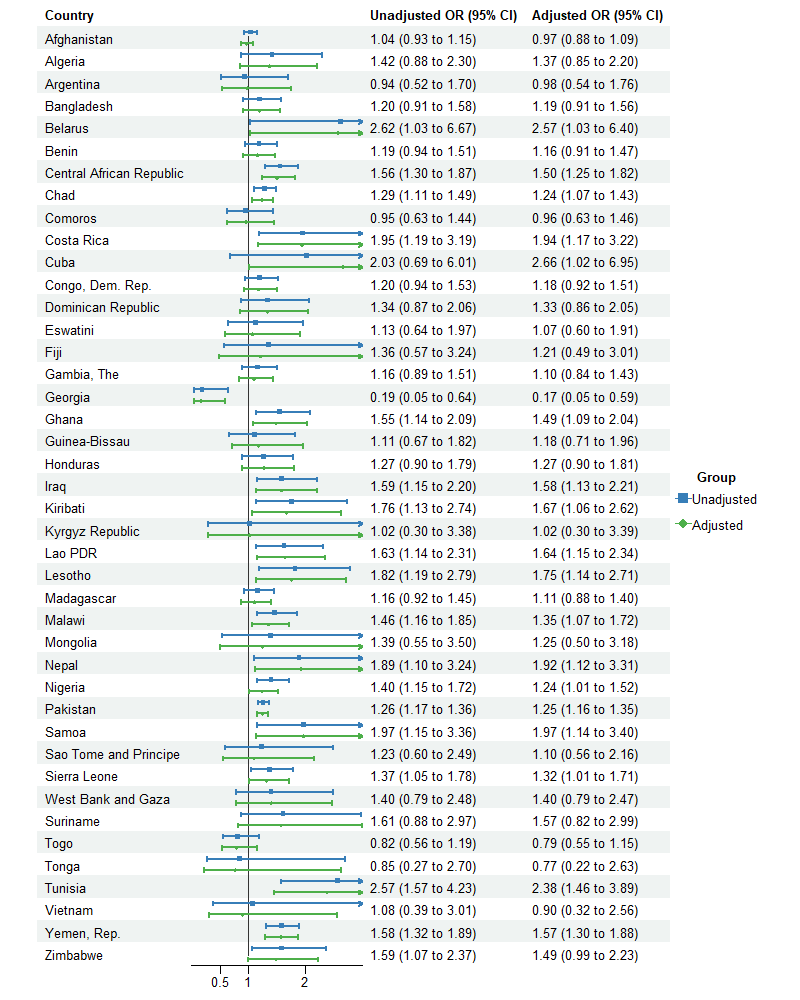


**Figure S3: Adjusted odds ratios for having fever disaggregated by country according to disability status in children aged 2–4 years from 42 Multiple Indicator Cluster Survey (MICS) countries**


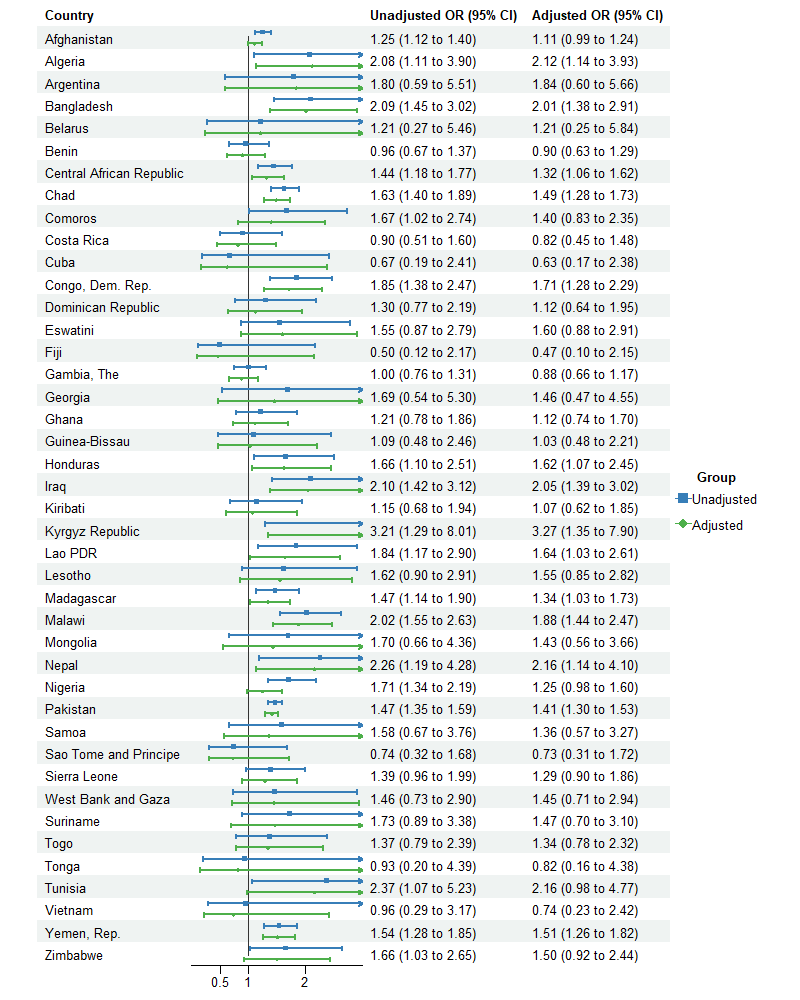


**Figure S4: Adjusted odds ratios for having diarrhea disaggregated by country according to disability status in children aged 2–4 years from 42 Multiple Indicator Cluster Survey (MICS) countries**

**Appendix 4**

**Table S5: Adjusted odds ratios for reported antibiotic use disaggregated by country economic status for Acute Respiratory Infection (ARI), diarrhea, and fever in children (2–4 years) with disabilities compared to those without disabilities**

|  |  | Reported antibiotic use for ARI | | Reported antibiotic use for diarrhea | | Reported antibiotic use for fever | |
| --- | --- | --- | --- | --- | --- | --- | --- |
|  |  | N (%) | aOR [95%CI]^a^ | N (%) | aOR [95%CI]^a^ | N (%) | aOR [95%CI]^a^ |
| Low income countries | Children without disabilities | 6,048 (41.7) | / | 1,732 (9.47) | / | 8,631(30.39) | / |
|  | Children with disabilities | 959 (42.25) | 0.97 [0.85,1.10] | 247 (8.94) | 0.90 [0.74,1.09] | 1,179 (32.17) | 1.00 [0.90,1.11] |
| Lower-middle income countries | Children without disabilities | 4,976 (43.31) | **^/^** | 1,937 (14.73) | / | 8,894 (31.79) | / |
|  | Children with disabilities | 504 (36.18) | **0.85 [0.74,0.97]** | 176 (11.59) | **0.78 [0.64,0.95]** | 783 (31.32) | 1.03 [0.93,1.15] |
| Upper-middle income countries | Children without disabilities | 851 (54.66) | / | 329 (19.47) | / | 2,544 (47.02) | / |
|  | Children with disabilities | 75 (58.14) | 1.31 [0.58,2.94] | 21 (17.07) | 1.29 [0.19,8.94] | 154 (47.98) | 1.35 [0.66,2.77] |


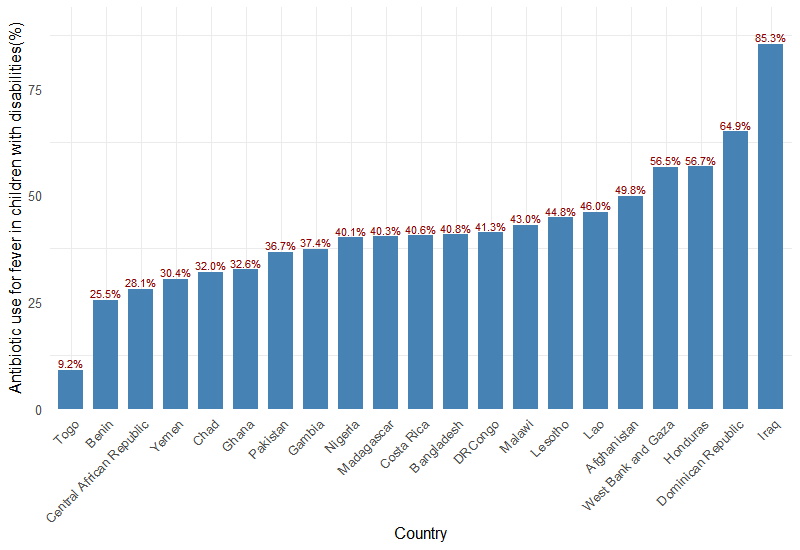


**Figure S5: Estimated proportion of reported antibiotic use for disabled children with ARI in the last 2 weeks**


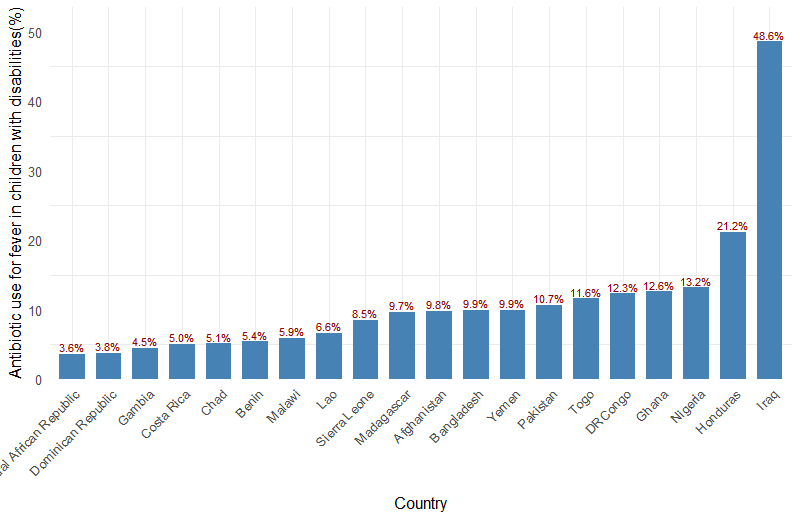


**Figure S6: Estimated proportion of reported antibiotic use for disabled children with diarrhea in the last 2 weeks**


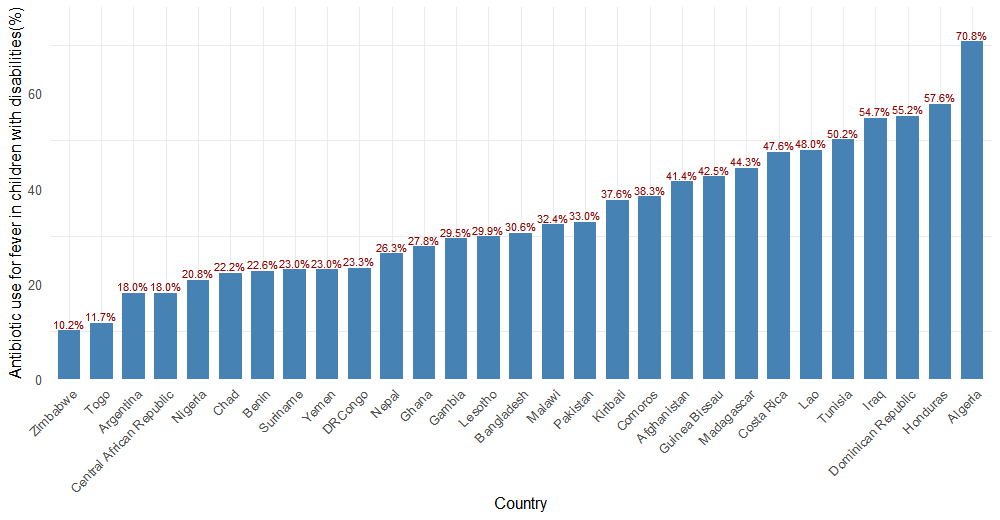


**Figure S7: Estimated proportion of reported antibiotic use for disabled children with fever in the last 2 weeks**

**Appendix 5: Sensitivity analysis**

**Table S6: Adjusted odds ratios for Acute Respiratory Infection (ARI), diarrhea, and fever in children (2–4 years) with disabilities compared to those without disabilities**

|  | Overall aOR^a^ | Girls with disabilities aOR^b^ | Boys with disabilities aOR^b^ |
| --- | --- | --- | --- |
| Having ARI | **1.79 [1.34,2.38]^c^** | **1.66 [1.20,2.30]^c^** | **1.87 [1.26,2.76]^c^** |
| Having Diarrhea | 1.15 [0.96,1.39] | 1.29 [0.98,1.69] | 1.07 [0.84,1.38] |
| Having Fever | **1.54 [1.21,1.96]^c^** | **1.36 [1.04,1.78]^c^** | **1.67 [1.19,2.34]^c^** |

^a Adjusted for country, age, sex, residence place, mother’s education and number of children under 5.^

^b Adjusted for country, age, residence place, mother’s education and number of children under 5.^

^c p<0.05^

**Table S7: Adjusted odds ratios for reported antibiotic use for Acute Respiratory Infection (ARI), diarrhea, and fever in children (2–4 years) with disabilities compared to those without disabilities**

|  | Overall aOR^a^ | Girls with disabilities aOR^b^ | Boys with disabilities aOR^b^ |
| --- | --- | --- | --- |
| Reported antibiotic use for ARI | 1.21 [0.75,1.94] | 0.67 [0.39,1.15] | 1.57 [0.85,2.92] |
| Reported antibiotic use for Diarrhoea | 0.94 [0.66,1.36] | **0.80 [0.65,0.98]^c^** | 1.02 [0.59,1.78] |
| Reported antibiotic use for Fever | 1.34 [0.89,2.02] | 0.83 [0.55,1.24] | 1.57 [0.91,2.73] |

^a^ Adjusted for country, age, sex, residence place, mother’s education and number of children under 5.

^b^ Adjusted for country, age, residence place, mother’s education and number of children under 5.

^c^ *p*<0.05

**Table S8: Adjusted odds ratios (aORs)for reported antibiotic use in children (2–4 years) with different types of disabilities compared to those without disabilities**

|  | Reported antibiotic use for ARI  aOR^a^ [95%CI] | Reported antibiotic use for diarrhea  aOR^a^ [95%CI] | Reported antibiotic use for fever aOR^a^ [95%CI] |
| --- | --- | --- | --- |
| Seeing | **0.67 [0.50,0.91]^b^** | 0.71 [0.46,1.11] | 1.05 [0.81,1.36] |
| Hearing | 0.95 [0.68,1.32] | 0.80 [0.50,1.27] | 0.79 [0.50,1.23] |
| Walking | 2.13 [0.69,6.58] | 0.89 [0.65,1.20] | 1.51 [0.60,3.81] |
| Controlling Behavior | 0.60 [0.34,1.06] | **0.69 [0.51,0.93]^b^** | 1.75 [0.86,3.60] |
| Playing | 1.20 [0.66,2.15] | 1.10 [0.83,1.46] | 1.34 [0.95,1.89] |
| Learning | 0.94 [0.54,1.66] | **0.77 [0.62,0.96]^b^** | 1.20 [0.69,2.08] |
| Communication | 1.58 [0.78,3.22] | 1.06 [0.49,2.30] | 1.08 [0.56,2.07] |
| Fine Motor Skills | 0.84 [0.52,1.36] | 0.97 [0.70,1.35] | 0.92 [0.71,1.19] |

^a^ Adjusted for country, age, sex, residence place, mother’s education and number of children under 5.

^b^ *p*<0.05

^
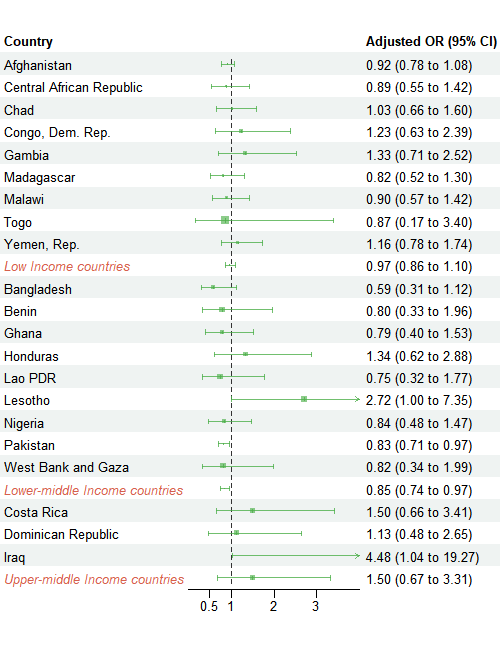
^

**Figure S8: Odds ratios (ORs) for reported antibiotic use for ARI in children (2–4 years) with disabilities compared to those without disabilities**

**
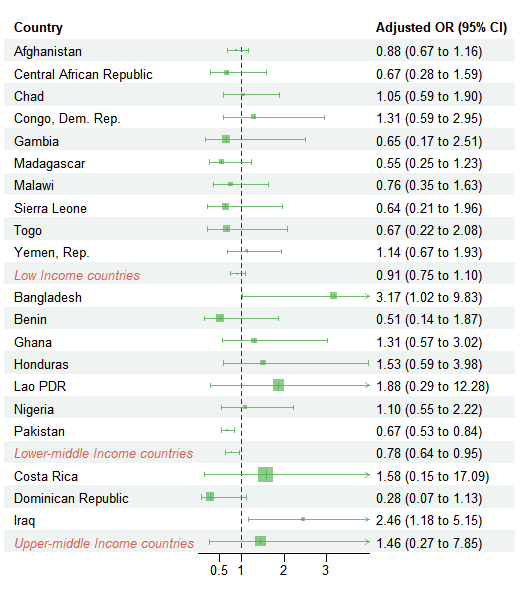
**

**Figure S9: Odds ratios (ORs) for reported antibiotic use for diarrhea in children (2–4 years) with disabilities compared to those without disabilities**

**
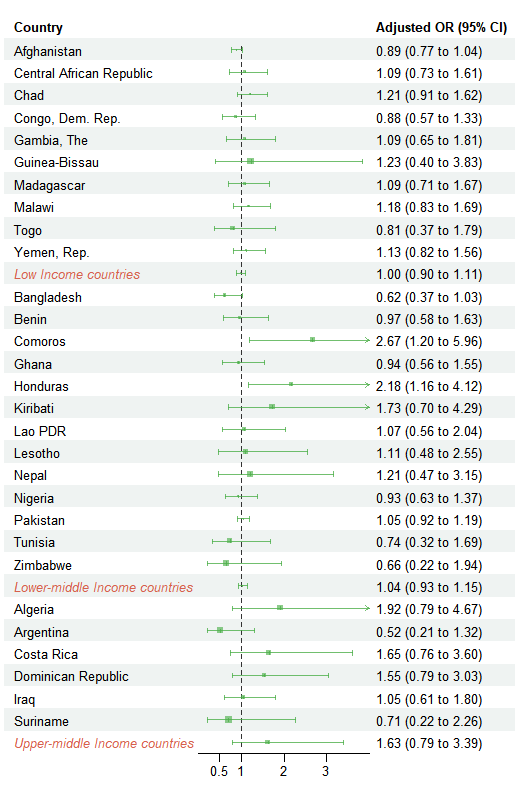
**

**Figure S10: Odds ratios (ORs) for reported antibiotic use for fever in children (2–4 years) with disabilities compared to those without disabilities**

**Appendix 6: Analysis removing the cases containing only negative and missing values in the eight domains of disabilities**

**Table S9: Adjusted odds ratios for having and reported antibiotic use for Acute Respiratory Infection (ARI), diarrhea, and fever in children (2–4 years) with disabilities compared to those without disabilities**

|  | Overall aOR^a^ | Girls with disabilities aOR^b^ | Boys with disabilities aOR^b^ |
| --- | --- | --- | --- |
| Having ARI | **1.84 [1.35,2.52]^c^** | **1.72 [1.19,2.47]^c^** | **1.94 [1.27,2.98]^c^** |
| Having Diarrhea | 1.17 [0.94,1.45] | 1.30 [0.94,1.81] | 1.08 [0.81,1.44] |
| Having Fever | **1.60 [1.24,2.08]^c^** | **1.42 [1.04,1.93]^c^** | **1.73 [1.20,2.49]^c^** |
| Reported antibiotic use for ARI | 1.17 [0.66,2.06] | 0.64 [0.32,1.26] | 1.50 [0.70,3.20] |
| Reported antibiotic use for Diarrhoea | 0.94 [0.58,1.54] | **0.72 [0.56,0.92]^c^** | 1.07 [0.52,2.21] |
| Reported antibiotic use for Fever | 1.28 [0.80,2.03] | 0.76 [0.43,1.32] | 1.56 [0.84,2.87] |

^a^ Adjusted for country, age, sex, residence place, mother’s education and number of children under 5.

^b^ Adjusted for country, age, residence place, mother’s education and number of children under 5.

^c^ p<0.05

**Table S10: Adjusted odds ratios (aORs) for reported antibiotic use in children (2–4 years) with different types of disabilities compared to those without disabilities**

|  | Reported antibiotic use for ARI  aOR^a^ [95%CI] | Reported antibiotic use for diarrhea  aOR^a^ [95%CI] | Reported antibiotic use for fever aOR^a^ [95%CI] |
| --- | --- | --- | --- |
| Seeing | **0.64 [0.46,0.90]^b^** | 0.76 [0.51,1.15] | 1.20 [0.91,1.58] |
| Hearing | 0.83 [0.55,1.25] | 0.91 [0.52,1.60] | 0.75 [0.44,1.27] |
| Walking | 2.42 [0.72,8.16] | 0.77 [0.53,1.11] | 1.55 [0.55,4.36] |
| Controlling Behavior | 0.57 [0.32,1.00] | **0.66 [0.48,0.92]^b^** | 1.56 [0.80,3.05] |
| Playing | 1.24 [0.52,2.93] | 1.08 [0.75,1.57] | 1.52 [0.91,2.53] |
| Learning | 0.81 [0.32,2.05] | **0.61 [0.45,0.82]^b^** | 1.33 [0.59,3.01] |
| Communication | 1.52 [0.66,3.47] | 1.22 [0.45,3.32] | 1.01 [0.49,2.09] |
| Fine Motor Skills | 0.69 [0.36,1.30] | 0.74 [0.49,1.11] | 0.85 [0.56,1.31] |

^a^ Adjusted for country, age, sex, residence place, mother’s education and number of children under 5.

^b^ *p*<0.05
